# Editorial: Insights in pediatric gastroenterology, hepatology & nutrition

**DOI:** 10.3389/fped.2025.1601516

**Published:** 2025-05-09

**Authors:** Andrew S. Day

**Affiliations:** Department of Paediatrics, University of Otago Christchurch, Christchurch, New Zealand

**Keywords:** bifidobacteria, pancreatitis, malrotation and volvulus, necrotizing enterocolitis (NEC), formula, premature infant

**Editorial on the Research Topic**
Insights in pediatric gastroenterology, hepatology & nutrition

## Introduction

The aim of this Research Topic was to gather a range of publications to illustrate some of the breadth of pediatric gastroenterology research in 2024. The six contributions touch on aspects such as the intestinal microbiome, nutrition in early life, pancreatitis and congenital anomalies leading to diarrhea.

A number of key factors are important in the development of the intestinal microbiome in early life (1). These include the method of birth delivery, early infant feeding practices and antibiotic exposure. The acquisition of the Bifidobacterium group/class is a key early stage in the development of the microbiome, with relevance to early nutrition and longer-term immune responses. Xu et al. evaluated the presence of Bifidobacteria in infants from the three predominant ethnicities in Singapore. Samples were collected from infants during the first 3 months of life. Real-time PCR was used to ascertain the presence and pattern of the bacteria of interest. Overall, less than 5% of the infants were found to have Bifidobacteria. Factors contributing to these patterns were immigration status, method of birth delivery and previous use of antibiotics.

Infants born prematurely are at increased risk for various adverse events, including compromised growth, gut disorders, chronic lung disease and poor neurodevelopmental outcomes. One gut disorder of note is necrotizing enterocolitis (NEC), which is more commonly seen in infants born prematurely than in those born at term. O'Connell provided an overview of key aspects of NEC and offered a new perspective on the approach to NEC.

Another aspect of the care of premature infants is the optimization of nutrition to ensure adequate growth: this may require enhancing the caloric content of the formula (2). Lavassini et al. prospectively compared the outcomes of a human milk-derived fortifier (HMDF) to those of a bovine milk-derived fortifier (BMDF) in a cohort of 139 premature infants. Specific outcomes of interest were growth and vitamin D status. Growth parameters at 4 and 8 weeks of age did not differ between the groups. However, those who received the HMDF had better vitamin D status at 4 weeks. Nevertheless, this study was not designed to elucidate the underlying mechanism behind this observation.

Baran et al. provided some interesting perspectives on changes in access to specific infant formulas in the United States in 2022. The global pandemic disrupted the supply of these formulas, with the peak disruption occurring in February 2022. The research team surveyed pediatric health care providers who managed children with cow's milk protein intolerance. The respondents' answers illustrated the impact of the formula shortage at that time upon the management of children.

In recent years a number of forms of recurrent or familial pancreatitis have been described: in a number of cases, the underlying genetic factors have been elucidated enabling a specific diagnosis to be made (3). In some instances, repeated episodes of acute pancreatitis may lead to chronic pancreatitis. Destro et al. reported the case of a young boy who was diagnosed with familial pancreatitis after his first episode of pain requiring hospitalization. This boy was also found to have a duplication of a portion of the pancreatic duct. The case report outlines his progress over time and then provides an overview and context for his issues.

The final article in this RT is another case report. Here, the authors described two infants who had presented with chronic intractable diarrhea and hypoalbuminemia (thought to be due to an associated protein-losing enteropathy). Numerous investigations were unhelpful and various interventions failed to improve the infants' condition. Finally, they were found to have small bowel malrotation with chronic volvulus. Correction of this anomaly led to the complete resolution of their problems.

Taken together, these contributions cover some varied and interesting aspects relevant to the field of pediatric gastroenterology. Hopefully, the publication of these articles will generate further research endeavors and also improve the outcomes for children suffering from these issues.
